# Cost of HPV screening at community health campaigns (CHCs) and health clinics in rural Kenya

**DOI:** 10.1186/s12913-018-3195-6

**Published:** 2018-05-25

**Authors:** Jennifer Shen, Easter Olwanda, James G. Kahn, Megan J. Huchko

**Affiliations:** 1 Philip R. Lee Institute for Health Policy Studies, University of California, 3333 California Street Suite 265, Box 0936, San Francisco, CA 94118 USA; 2 Duke Global Health Institute, Duke University, Durham, NC USA; 3 Department of Obstetrics and Gynecology, Duke University, Durham, NC USA; 4 Center for Microbiology Research, Kenya Medical Research Institute, Nairobi, Kenya

**Keywords:** Cervical cancer screening, HPV testing, Micro-costing, Rural Kenya

## Abstract

**Background:**

Cervical cancer is the most frequent neoplasm among Kenyan women, with 4800 diagnoses and 2400 deaths per year. One reason is an extremely low rate of screening through pap smears, at 13.8% in 2014. Knowing the costs of screening will help planners and policymakers design, implement, and scale programs.

**Methods:**

We conducted HPV-based cervical cancer screening via self-collection in 12 communities in rural Migori County, Kenya. Six communities were randomized to community health campaigns (CHCs), and six to screening at government clinics. All HPV-positive women were referred for cryotherapy at Migori County Hospital. We prospectively estimated direct costs from the health system perspective, using micro-costing methods. Cost data were extracted from expenditure records, staff interviews, and time and motion logs. Total costs per woman screening included three activities: outreach, HPV-based screening, and notification. Types of inputs include personnel, recurrent goods, capital goods, and services. We costed potential changes to implementation for scaling.

**Results:**

From January to September 2016, 2899 women were screened in CHCs and 2042 in clinics. Each CHC lasted for 30 working days, 10 days each for outreach, screening, and notification. The mean cost per woman screened was $25.00 for CHCs [median: $25.09; Range: $22.06-30.21] and $29.56 for clinics [$28.90; $25.27-37.08]. Clinics had higher costs than CHCs for personnel ($14.27 vs. $11.26) and capital ($5.55 vs. $2.80). Screening costs were higher for clinics at $21.84, compared to $17.48 for CHCs. In contrast, CHCs had higher outreach costs ($3.34 vs. $0.17). After modeling a reduction in staffing, clinic per-screening costs ($25.69) were approximately equivalent to CHCs.

**Conclusions:**

HPV-based cervical cancer screening through community health campaigns achieved lower costs per woman screened, compared to screening at clinics. Periodic high-volume CHCs appear to be a viable low-cost strategy for implementing cervical cancer screening.

**Electronic supplementary material:**

The online version of this article (10.1186/s12913-018-3195-6) contains supplementary material, which is available to authorized users.

## Background

### Introduction

Invasive cervical cancer is highly preventable through organized screening programs. However, globally over half a million women are diagnosed with the disease each year [1]. Cervical cancer is the most common cancer among women in Sub-Saharan Africa [2]. Nine out of 10 cervical cancer deaths occur in low-resource countries, with an 85% mortality rate in Sub-Saharan Africa [3, 4]. While in developed countries a major decline in cervical cancer incidence and mortality occurred after the introduction of cytologic testing, many developing countries still lack effective screening programs [5].

High cancer mortality rates in developing countries may be due to inadequate health facilities and lack of personnel [6–8]. Organized screening programs are rare, and screening rates are low in developing countries, where only 19% of women are screened for cervical cancer as compared to 83% in the U.S. [9]. Simplified screening strategies employing testing for high-risk human papillomavirus (HPV), the causative agent in most cervical cancers, have been shown to reduce mortality from cervical cancer when coupled with outpatient treatment of precancerous lesions [5, 10, 11]. Programs implementing HPV testing in community-based settings may address the infrastructure and personnel limitations that prevent screening in some low-resource settings.

In Kenya, cervical cancer is the most frequent cancer among women, with about 4800 annual diagnoses and over 2400 deaths per year [12]. HPV testing is recommended by Kenya’s Ministry of Public Health and Sanitation’s Division of Reproductive Health, but is not reliably incorporated into most community or government programs [13]. Visual inspection with acetic acid-based screening is available at some clinics in Kenya. Screening rates are extremely low, at 13.8% nationwide and below 11% in rural areas in 2014 [14]. Even when screening is available, uptake can remain poor, due to low levels of knowledge of cervical cancer [15], and lack of awareness of HPV screening and the risks of cervical cancer [16]. Very few women recognize that routine testing is the primary way to prevent cervical cancer [17].

The community-based health fair is an important strategy to help people in developing countries access important preventive healthcare messaging and services. Community health campaigns (CHCs) occur over a short duration, delivering preventive health services at high-volume close to residential areas. CHCs have been effective in reducing maternal and neonatal mortality [18–20], prevention of malaria [21], HIV testing and delivery of antiretrovirals [22, 23], tuberculosis detection [24], and increasing mental health care access [25]. A multi-disease CHC in rural Kenya found increased ART coverage and other health benefits with cost savings [26, 27]. CHCs are part of a community-based healthcare model, which has gained traction in recent years because it can mobilize a large proportion of a community, and may be less resource-intensive than receiving preventive care at clinics.

In this study, we used costing data collected during a cluster-randomized trial in rural Kenya comparing uptake of HPV-based cervical cancer screening in community health campaigns and government clinics to compare the costs of the strategies. This is the first study to estimate the costs of cervical cancer screening with a community-based health campaign strategy, and furthermore, compares the costs of two cervical cancer screening interventions in Kenya. The cost estimations will help inform future implementers on funding and resource needs for cervical cancer screening.

### Study description

This study was conducted as part of a cluster-randomized trial in 12 rural communities in Migori County in the former Nyanza Province of western Kenya between January and September 2016. Two-thirds of citizens of Nyanza live on less than $1 per day, and the province has the highest prevalence of HIV in the country. The target population was women who were eligible for and would benefit from cervical cancer screening per the Kenya Ministry of Health Guidelines: 25 to 65 years old with an intact uterus and cervix. Site selection was based on census data, health facility information, mapping, and demographic data [28]. Each community was a cluster of villages or sub-locations within a defined administrative boundary. The twelve communities were randomized in a 1:1 ratio using an allocation sequence generated by Stata 11 MP (StataCorp, TX). Six communities were randomized to a CHC intervention and six to HPV screening at government clinics.^1^ Despite random assignment, women in CHCs were slightly older and more likely to have been screened for cervical cancer in the past [28]. Non-adjacent communities with a population of 4500 to 9000, with at least one health facility were identified. Both arms included three phases for which costs are presented: outreach and mobilization (“Outreach”), HPV-based screening (“Screening”), and notification of results and standard referral (“Notification”). Treatment services were equivalent between the arms, and therefore not costed. More information on the details of the study design can be found in the main outcomes paper, Huchko et al. 2017 [28].

**Fig. 1:**
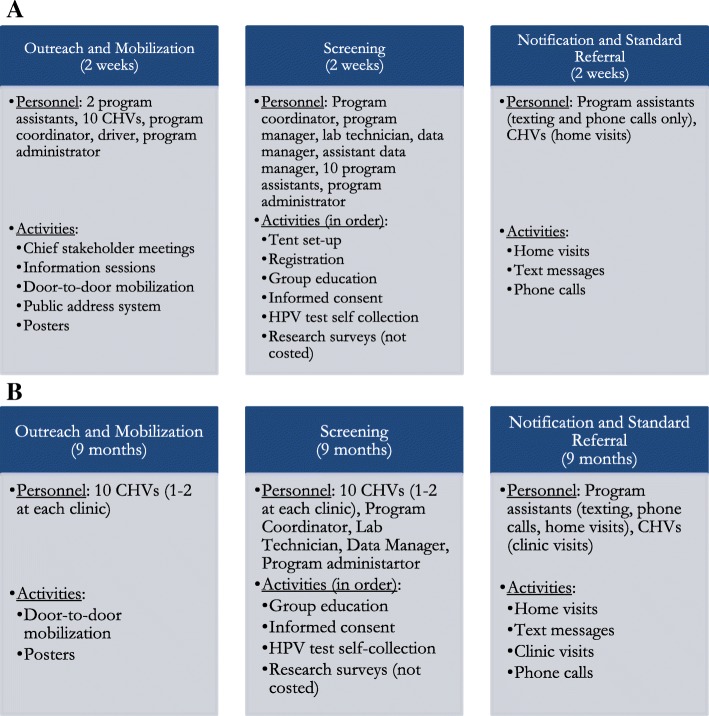
**a** Personnel team and activities of CHCs across 3 phases: outreach, screening, and notification. **b** Personnel team and activities of Clinics across 3 phases: outreach, screening, and notification

Figure 1a and b summarize the workflows for each of the three phases for CHC communities and clinic communities, respectively. For CHC communities, each of the three phases (Outreach, Screening, and Notification) lasted two weeks. A subset of the CHC team conducted outreach through stakeholder meetings, information sessions, door-to-door mobilization, announcements using a public-address system, and posters. During screening, the entire CHC team traveled to different areas of the CHC community every morning and set up tents. The team then conducted a sequence of activities dedicated to screening for each woman visiting the tents: registration, group education, informed consent, and self-collection of screening specimens using the careHPV test kit. The lab technician then tested whether the woman was HPV-positive using the *care*HPV™ test system. After two weeks of screening activities, the CHC team moved onto notification of results and standard referral, which again lasted two weeks. Program assistants worked on using text messages and phone calls to notify women of their results, and Community Health Volunteers (CHVs) employed by the program conducted door-to-door home visits to notify women of their status. HPV-positive participants received referral to a treatment site located at Migori County Hospital.

In contrast to CHCs, each of the three phases were conducted concurrently at the six clinic communities for nine months total. After an initial series of key stakeholder meetings and information sessions prior to launching screening, one to two CHVs at each clinic conducted outreach through door-to-door mobilization and posters. During screening, CHVs, with supervision from the program coordinator and assistance from the data manager and program administrator, conducted all the screening activities at the clinic sites. They conducted group education, acquired informed consent, and collected HPV self-collection test kits. Notification also occurred throughout the nine months of screening at each clinic site. Since CHVs attended clinics fulltime and were in charge of notifying women who decided to visit clinics to receive test results, the CHVs were not involved with home visit notification, in contrast to CHCs. Instead, a team of program assistants would conduct home visits (1-3 days per month for each clinic) to notify women of their test results. The program assistants issued text messages and phone calls. HPV-positive women were referred for treatment at Migori County Hospital. More detailed information on the CHC and clinic community workflows is in the Additional file 1.

## Methods

Micro-costing procedures, a method of valuation in health economics that involves the “direct enumeration and costing of every input consumed in the treatment of a particular patient” (Siegel et al. 1996), were applied to quantify resources used for HPV testing in each arm (clinic and CHC). Recommendations on economic evaluations of health system interventions made by Drummond and Jefferson (1996) and Weinstein et al. (1996) guided our analyses [29, 30]. Direct costs were estimated from the health system perspective through a prospective analysis. We conducted an economic analysis of costs, where all inputs were costed, whether donated or subsidized, and costs of labor or wage rates were based on market wages and salaries. The micro-costing method was a hybrid of top-down and bottom-up, depending on the cost-item and data collected. For most cost items, we enumerated the item (based on numbers used for the community), multiplied the number by the cost of each item whether purchased or donated, and estimated unit (per-screening) costs from the sum of costs.

Capital goods cost estimation was based on total costs from expenditure records. Capital goods are tangible assets including vehicle for transportation, tents for CHCs, and the *care*HPV ™ test system, which is a rapid batch diagnostic test designed for HPV-testing in low-resources settings (Qiagen, Cost Gaithersburg, Maryland). Costs of capital items were amortized on a straight-line basis over five years assuming no salvage value. For the vehicle and tents, the costs were allocated evenly across the 6 CHC communities. For the *care*HPV ™ test system, costs were allocated on a per-screening basis in each of the 12 communities. Communities with more screenings had higher costs allocated for the test system. More information on capital goods costing is in the Additional file 2.

Personnel costs were estimated based on salary records. Costs of personnel for CHCs were based on the monthly salaries and the amount of time spent on each phase of the CHC. Since all phases for CHCs lasted two weeks, the personnel cost for a phase was the personnel’s estimated salary for two weeks. Since clinic personnel worked on all six clinic communities concurrently, the total per-clinic personnel costs were estimated based on the number of women screened at each clinic, i.e. the proportion of women screened in each clinic community over the total number of women screened at clinics. More information on personnel costing is in the Additional file 2.

The primary data used to measure the costs of each intervention activity were expenditure records, interviews, time and motion logs, direct counts made by the costing lead, market rates, salary records, and estimations based on government data (MOH/NASCOP) for facility space costs. Tables 1 and 2 of the (Additional file 3: Table S1 and Additional file 4: Table S2) show in-depth information on the data sources for quantity, price, and percent allocation towards program or non-program activities at CHC and clinic communities. The cost data was collected manually and subsequently electronically recorded on Excel workbooks. The cost items were classified under five input types: personnel, recurrent goods, services, capital goods, and facility overhead. The number of units and price of each input was recorded. Number of input units and price were converted into a total economic cost. All costs reported are in 2016 U.S. dollars, converted from Kenyan shillings at an exchange rate of 95 Kenyan shillings per U.S. dollar [31].

**Table 1:** Cost estimations, in 2016 USD, by phase and cost type, per woman screened with self-collected HPV in community health campaigns in six communities in 2016

	Community Health Campaign Community #					
	1	2	3	4	5	6
Outreach						
Capital Goods	0.51	0.92	0.67	0.72	0.64	0.53
Personnel	2.00	3.18	2.32	2.26	1.66	1.18
Recurrent Goods	0.01	0.02	0.02	0.56	0.24	0.25
Services	0.32	0.51	0.35	0.40	0.43	1.01
Outreach Subtotal	2.84	4.63	3.36	3.94	2.97	2.97
Screening						
Capital Goods	1.18	1.82	1.38	1.47	1.59	1.15
Personnel	5.09	9.00	7.72	6.40	4.91	4.80
Recurrent Goods	8.11	8.82	8.42	8.70	8.21	8.09
Services	2.36	2.31	1.16	1.48	1.30	1.46
Screening Subtotal	16.74	21.95	18.69	18.05	16.02	15.51
Notification						
Capital Goods	0.51	0.92	0.67	0.72	0.64	0.53
Personnel	3.47	2.17	3.47	3.28	3.66	2.78
Recurrent Goods	0.08	0.02	0.08	0.07	0.04	0.05
Services	0.33	0.51	0.12	0.13	0.48	0.22
Notification Subtotal	4.40	3.63	4.34	4.21	4.82	3.58
Women screened	602	337	461	430	483	586
Cost per screening	23.98	30.21	26.39	26.21	23.81	22.06

**Table 2:** Cost estimations, in 2016 USD, by phase and cost type, per woman screened with self-collected HPV in clinics, in six communities in 2016

	Clinic Community #					
	1	2	3	4	5	6
Outreach						
Personnel	0.10	0.19	0.19	0.14	0.12	0.20
Recurrent Goods	0.01	0.02	0.02	0.01	0.01	0.02
Outreach Subtotal	0.11	0.21	0.21	0.15	0.13	0.22
Screening						
Capital Goods	1.89	2.20	1.33	1.66	2.09	3.57
Facility	10.78	2.49	3.32	2.17	0.41	2.09
Personnel	8.10	6.72	6.63	6.63	6.75	6.83
Recurrent Goods	7.24	7.73	6.43	6.86	7.98	9.70
Services	1.70	2.04	2.28	1.84	1.55	2.82
Screening Subtotal	29.73	21.17	19.99	19.17	18.78	25.01
Notification and Standard Referral						
Personnel	5.82	8.00	7.96	6.85	6.31	8.29
Services	1.42	0.26	0.00	0.09	0.05	0.00
Notification Subtotal	7.24	8.26	7.96	6.94	6.35	8.30
# women screened	326	502	338	450	269	157
Cost per screening	37.08	29.64	28.16	26.26	25.27	33.53

Notes: Community #2 had a total of 3 separate clinics; Community #3 had a total of 2 separate clinics; and Community #4 had a total of 2 separate clinics

Once total economic cost of each item was calculated, each cost item was further allocated to program and non-program purposes. For example, time spent on administering pre- and post-screening surveys was a research activity, and was omitted from program cost estimates. To arrive at a unit cost (per completed screening), total economic cost of each item that was designated for program purposes was divided by the number of women screened at each community. The Additional file 2 provides further detail on how each category of cost (personnel, capital goods, facility, recurrent goods, services) was recorded and estimated.

Using the unit-cost estimations, the micro-costing data was then aggregated to estimate total costs per woman screened at a CHC and clinic community. In the costing analyses, we compared costs across the six CHC communities, and conducted a similar comparison across the six clinic communities. The costs per woman screened at each of the 12 communities were broken down by type of costing input (personnel, facility, services, recurrent goods, capital), and phase (outreach, screening, and notification). We did not include cost of treatment because the treatment model was identical between the CHC and clinic arms.

### Sensitivity analysis

A series of sensitivity analyses were conducted to assess whether costs per screening estimated are sensitive to changes in implementation. First, cost estimates for CHCs were conducted under two alternate transportation scenarios to purchasing a program vehicle: (1) issuing travel reimbursements, or (2) renting a vehicle. For two of the six CHCs, travel vouchers were issued because a vehicle was not purchased at the time of the CHC. Travel reimbursement rates for these two CHCs were therefore used to approximate cost per kilometer of travel.^2^ A rented vehicle costs 4000 KES per day, or 42 USD a day.

In the second sensitivity analysis, we explored a scenario where clinic personnel are used more efficiently, and therefore measure the costs of reducing the number of personnel dedicated to clinic screening. The research team, based on observation and experience, believed that each clinic could have hired another CHV who was dedicated to conducting home visits for the notification phase, rather than using program assistants. During the study, clinics each had five program assistants conduct home visits 1-3 times a month, which may have driven up personnel costs for notification at clinics. The team also believed that the program coordinator and data manager could have reduced their time spent on clinic activities, and delegated more responsibility to a program assistant, who would manage operations and data collection. The maximum number of clinics a program assistant could have managed was two clinics. Therefore, in the sensitivity analysis, a program assistant would dedicate full time to monitoring screening and notification at two clinics, where he or she would oversee the progress of the CHV and make appointments with certain patients who need more attention. The program coordinator would commit 5% time to each clinic, and lab technicians would dedicate time to each clinic based on the number of women screened (same as before, average 10% time across clinics). Each clinic would therefore only have four team members: CHV, program assistant, program coordinator, and lab technician. The personnel ratio per clinic would reduce from 2 to 1.65.

Finally, we also implemented cost regressions to estimate the fixed cost for the screening program and the marginal costs of each screening. We estimated total cervical cancer screening costs, one from each community, and regressed total costs on numbers of women screened in each community. Each cost regression has 6 observations, one from each CHC and clinic community.

## Results

Between January and September 2016, 2899 women were screened at the six CHC communities and 2042 at the six clinic communities. In Tables 1 and 2, the site-by-site cost breakdown per woman screened is presented. The mean cost per woman screened was $25.00 for CHCs and $29.56 for clinics. The mean number of women screened was 482 per CHC and 340 per clinic.^3^

### Differences between CHCs and clinics

**Fig. 2:**
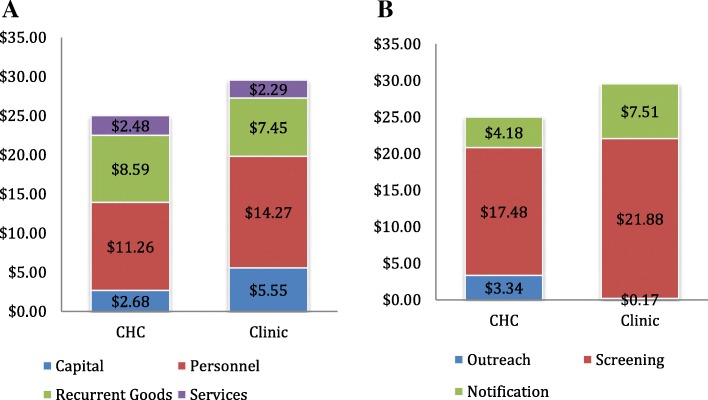
**a** and **b** CHC and clinic average cost per screening estimates, color coded by cost type and phase

Figure 2a and b depict cost breakdowns of CHC and clinic community costs, by cost type and phase, respectively. Personnel costs were higher for clinics ($14.27 per screening) compared to CHCs ($11.26 per screening). Capital costs were also higher for clinics ($5.55) compared to CHCs ($2.68), due to the need for clinics to pay for renting space at facilities.^4^ Services were almost equivalent ($2.48 for CHCs and $2.29 for clinics). Recurrent goods were the only input type that was slightly higher for CHCs, at $8.59 per screening compared to $7.45 for clinics; this reflects additional transportation costs associated with executing a CHC (e.g. fuel costs and transport reimbursements).

When comparing CHCs and clinics across activities, per-screening costs were higher for CHCs only in outreach ($3.34 vs $0.17 at clinics), whereas per-screening costs were higher for clinics for screening ($17.59 for CHCs and $21.88 for clinics) and notification ($4.18 for CHCs and $7.51 for clinics). CHCs had higher costs for outreach because they conducted more intensive outreach activities than clinics, including door-to-door mobilization, public address systems, and meetings with chief elders. A larger team of personnel was involved with CHC outreach activities. At clinics, the only outreach conducted was door-to-door mobilization by CHVs. During door-to-door mobilization at clinics, CHVs visited homes one-by-one and provided information on cervical cancer screening conducted at clinics. Costs of mobilization were low for clinics because daily compensation of CHVs is lower than wage rates for personnel staff. In clinic communities, CHVs also put up posters with further information about screening. The outreach activities at CHCs generated higher numbers of women screened at CHCs compared to clinics (483 vs. 340) in a shorter period of time (10 days vs. nine months).

**Fig. 3:**
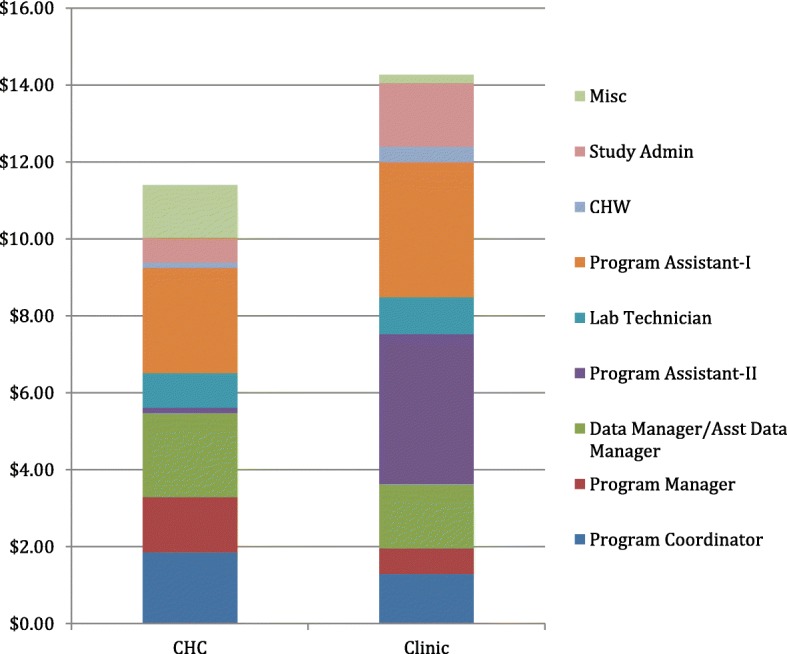
Bar graph of CHC and clinic personnel cost estimate breakdowns, by cadre

Figure 3 shows further breakdown of personnel costs by cadre. While CHCs had higher costs for data management and other miscellaneous personnel costs (e.g. driver, security, tent setup team), clinics had higher personnel costs associated with hired program assistants (type 1 was involved with monitoring screening at the clinics, and type 2 were involved with home visits during notification).

### Differences across sites, for CHCs and clinics

Variation in costs per woman screened across CHCs can largely be attributed to differences in numbers of women screened at each CHC. For example, personnel team size and compensation rates do not change much across CHCs, but per-screening personnel costs for the screening phase among CHCs range from $4.80 to $9.00. Similarly, for capital goods, the cost of capital goods such as the careHPV test system, tablet, and pipettes, do not change for different CHC communities. Per-screening capital goods costs for the screening phase therefore vary because of differences in numbers of women screened at each community. Variation in recurrent goods ($8.09 to $8.82 per screening during the screening phase) occurred across communities because of difference in fuel costs and amount of supplies required for screening. Variation in services offered for notification (texts, calls, and home visits) occurred because of differences in notification options selected at each CHC.

Variation in cost per screening for clinics was primarily due to differences in costs of facility space. For most clinics, facility rent was not entirely allocated for cervical cancer screening, because other health services such as HIV testing were conducted in the same space. One clinic, however, had considerably higher facility costs because 100% of a clinic room was allocated to CCSP screening. Clinic personnel designated to screening activities shifted across the six clinics, which is why there was slight variation in clinic-level personnel costs. The team of personnel involved with notification did not change across clinics, and therefore, personnel costs for notification did not vary.

For capital goods, all six communities used the central careHPV Test System and shared two motorbikes. Similar to personnel, clinic-level costs of capital goods were estimated based on proportion of women screened at each clinic, and therefore did not vary much across the clinics.

### Sensitivity analyses

In the sensitivity analysis in which transportation strategies were varied, the average cost per screening for CHCs under both transportation scenarios declined very slightly, at $23.12 per screening with a range of $20.53 to $27.51 for the transport reimbursement scenario, and at $24.98 per screening with a range of $21.68 to $30.42 for the rented vehicle scenario. The CHC cost per screening estimates are therefore not very sensitive to changes in transportation type used for the program.

In the second sensitivity analysis, personnel costs at clinics were estimated with an adjustment to team structure. During the program, a total of 11 individuals comprised clinic personnel: eight working on screening, and nine working on notification, with a personnel ratio of about two per clinic. Additionally, each clinic had one CHV working on both screening (trained to help women self-sample using HPV self-test kits) and notification.

Table 3 shows the results of the sensitivity analysis. Average cost per screening reduced to $25.69 for clinics, with a range of $20.57 to $32.12. The results measured a change in personnel efficiency. The average cost per screening from the sensitivity analysis was similar to per-screening costs of CHCs, differing by only 69 cents.

**Table 3:** Sensitivity analysis with modeled costs per screening based on a reduction in personnel responsible for screening and notification at clinics

Clinic Community #	Observed Clinic Costs (from Table 2)	Per-screening costs at each clinic after personnel adjustment
1	$37.08	$30.93
2	$29.64	$26.62
3	$28.16	$24.89
4	$26.26	$22.27
5	$25.27	$20.57
6	$33.53	$32.12
Average cost per screening	$29.56	$25.69

Notes: Estimated per-screening costs at each of the 6 control clinics based on a reduction in number of personnel involved with screening and notification. Each clinic is designated 1 CHV dedicated to both screening and notification. One program assistant dedicates 100% of their time to monitoring screening and notification at 2 clinics. The program coordinator will commit 5% time to each clinic, and lab technicians dedicate time to each clinic based on the number of women screened (same as before, average 10% time). Each clinic will therefore only have 4 team members: CHV, HE, program coordinator, and lab technician. The personnel ratio per clinic will reduce from 2 to 1.65. No other changes were made to estimated costs

Finally, we conducted two cost regressions for the six CHCs and six clinic communities to empirically estimate the program fixed cost per community screened and the marginal cost of a screening. For CHCs, the regression results showed an intercept of $5368.24 representing fixed costs, and marginal costs of $13.89, representing variable costs. For clinic communities, the regression results showed an intercept of $982.27 representing fixed costs, and marginal costs of $26.68, representing variable costs. CHCs had higher fixed costs than clinics, and clinics had higher marginal costs than CHCs.

## Discussion

This study determined and compared the costs of HPV based cervical cancer screening for women in Kenya through two distinct strategies: HPV self-sampling offered in brief community health campaigns (CHC) designed to rapidly screen women, or in government clinics. The study found that the CHC was less expensive ($25.00 per screening) than HPV based-screening at government clinics ($29.56 per screening), at a difference of $4.56 per screening. The findings can help inform the planning and design of future cervical cancer screening programs implemented in similar low- and middle-income regions with high-burden of disease. Etiologies of increased costs throughout the cervical cancer prevention cascade can help identify areas to target and develop more cost-efficient strategies for future evaluation.

Costs per screening differed between CHCs and clinics for two main reasons. First, CHCs may have had lower per-screening costs than clinics because of higher numbers of women screened on average in CHC communities. CHCs were likely more successful at recruiting women for cervical cancer screening because of more intensive outreach and mobilization efforts. Outreach and mobilization for CHCs involved a larger team than clinics, and included several more high-visibility promotion activities including involvement of community elders and public-address systems. Since CHCs screening activities were placed in prominent locations within each community, the excitement surrounding a community health “event” may have also encouraged more women to get screened. The more intensive recruitment efforts dedicated to CHCs brings up the question of whether more intensive recruitment for government clinics would have led to more screenings in control communities. The CHC was mobile, switched locations each day of the 10-day campaign, and was more integrated with communities. Thus, it is difficult to attribute the higher numbers of women screened to simply more outreach, or the combination of outreach and CHC integration and visibility within communities. Future studies could incorporate equivalent outreach efforts for government health clinics to identify the effect of a screening site that is mobile and integrated within the community.

The second possible reason for higher costs per screening in clinics is that there was too much staffing allocated for clinics during the study, especially with the lower numbers of women screened. Given the possibility that personnel size for implementation at clinics was larger than necessary, we simulated costs under the scenario of a smaller team dedicated to clinic screening activities (18% decline in personnel) in the sensitivity analysis. For this hypothetical scenario, average costs per screening were approximately the same as average costs per screening at CHCs ($25.69 versus $25.00). It is difficult to conclude that all other activities at the clinic, including the number of women who visited the clinic for screening, would have been exactly equivalent under this hypothetical personnel structure in the sensitivity analysis. Future studies could explore effectiveness of clinic screening with fewer personnel involved with screening and notification, and the cost models are helpful adjuncts to discussions with program planners.

This was the first study to measure costs of self-sampling in Kenya using cost data captured directly from program implementation, including not just the screening, but the essential mobilization and notification components. Furthermore, this study was the first to compare the costs of two different implementation strategies for cervical cancer screening in Kenya, a low income, and high-burden country. Prior to this study, cervical cancer costs in Kenya were only measured using a predictive model [32] and through a time and motion study of integrating cervical cancer screening at one HIV clinic [33]. The costs of implementation were comprehensively estimated through collection of program data, which can be used as reference for future cervical cancer screening programs in Kenya.

This analysis had several limitations. The first is the low number of communities where CHCs were implemented; Six CHC communities were compared with six control communities. Including more communities in the study may have allowed for more robust comparisons in costs per screening. The second, an efficiency curve occurred for CHCs that may have caused the first campaigns to be more expensive. The final consideration is whether CHCs can feasibly be scaled-up as a nationwide program, and whether government or donor support could fund regular CHCs.

## Conclusion

This study compared six short-term cervical cancer screening community health campaigns with six communities offering cervical cancer screening at clinics in rural Kenya. Results show that mean costs per woman screened at the six CHC communities were slightly lower than mean costs per woman screened at the six clinic communities ($25.00 compared to $29.56). In our experience, cervical cancer screening at community health campaigns increased screening coverage and lowered per-women screening costs as a result. Future cervical cancer screening programs should consider the option to integrate screening activities at community health campaigns, with an emphasis on increasing the efficacy of outreach to increase screening numbers and improve efficiency.

## Additional files

Additional file 1: Detailed information on CHC and Clinic Workflow: Includes in-depth information on how screening was set-up and facilitated by the implementation team and providers at both CHCs and clinics. (DOCX 17 kb)

Additional file 2: Further detail on cost estimation methods of each cost type (personnel, capital, facility, recurrent goods, and services): Includes in-depth information on the sources, data collection method, and cost estimation method of each of the major cost category types. Includes how personnel costs were allocated across sites, and how capital costs were amortized. (DOCX 16 kb)

Additional file 3: **Table S1.** Major cost items for each cost type for CHCs, with data source information on quantity, unit and total costs, and % allocation across purposes. (DOCX 19 kb)

Additional file 4: **Table S2.** Major cost items for each cost type for Clinics, with data source information on quantity, unit and total costs, and % allocation across purposes. (DOCX 18 kb)

Additional file 5: **Table S3.** Cost estimations per woman screened at all 10 clinics, from January to September 2016. Includes cost estimations of the three phases of implementation: outreach and mobilization, screening, and notification and standard referral. (DOCX 22 kb)

## Abbreviations

CHC: Community health campaign
CHV: Community health volunteer
HPV: Human papillomavirus

## Footnotes

1 The 6 CHC communities were Oyani, Ongito, Nyarongi, Nyamanga, Obware, and Ageng’a. The 6 clinic communities were Diruma, God Jope, Got Kachola, Nyamasare, Ondong, and Winjo. There were 6 communities where HPV screening was conducted through clinics, but a total of 10 clinics.

2 We assume that each liter of gasoline translates to 96 Kenyan shillings.

3 The costing table for each of the 10 clinics is in the Additional file 5: Table S3.

4 In this figure, facility costs incurred at clinics were labeled as a capital cost, since rent represents payment towards a capital investment.
