# Supplementary material for: Cost of HPV screening at community health campaigns (CHCs) and health clinics in rural Kenya
Source: BMC Health Serv Res. 2018 May 25;18:378. doi: 10.1186/s12913-018-3195-6 (PMC5970469; doi:10.1186/s12913-018-3195-6)
Supplement: Supplementary file 1 — Detailed information on CHC and Clinic Workflow: Includes in-depth information on how screening was set-up and facilitated by the implementation team and providers at both CHCs and clinics. (DOCX 17 kb) [file 12913_2018_3195_MOESM1_ESM.docx]

**Additional file 1**

T.S. Section 1: Detailed information on CHC and Clinic Workflow

*CHC Workflow*

In the CHC model, each activity (Outreach, Screening, and Notification) lasted for two weeks, so the program was in each community for a total of six weeks. Two program assistants and 10 community health volunteers (CHVs) carried out CHC Outreach. Outreach included stakeholder meetings with chiefs; information sessions at markets, churches, and women’s group meetings; and door-to-door mobilization, posters, and a public address system. The team provided information about dates, location of screening activities, and eligibility (ages 25 to 65 with intact uterus and cervix).

Mobile CHC screening was held at up to 10 sites per community over a period of two weeks. Each day, a team would travel to a new site and set up the tents, tables, and chairs. Sites for the mobile CHC included church compounds, schools, open fields, and market centers. The tent had a partitioned area for self-collection, a registration table, and a group education area. During the day, CHVs would conduct door-to-door mobilization under the supervision of two program assistants. Women interested in screening would arrive at the tent, register, and participate in a group education and consent process. After signing the individual consent form and completing a short pre-test survey at the pre-screening station, women were directed to the partitioned areas to self-collect specimens for HPV testing, using a pre-packaged kit with visual aids to help them. After submitting a sample, women completed a brief post-collection survey conducted at the post-test station. Samples were then taken to the laboratory at Migori County Hospital for processing and batch analysis with the careHPV system, which provides a result of positive if at least one of 14 high-risk genotypes of HPV is identified.

Personnel for CHC screening comprised a team of 16 people. The program coordinator oversaw the activities of the CHC and ensured supplies were adequate and ensured timely collection and testing of samples. The program manager helped the program coordinator and assisted in all tasks required for the smooth functioning of the CHC. The lab technician occasionally attended the CHCs to perform quality control checks on the sample collection process, and packaging and transportation of commodities. When not at the CHCs, the technician processed the samples at Migori County hospital. The data manager and assistant supervised the program assistants on the data collection process and handled all data issues. There were a total of 10 program assistants: two for mobilization, two for registration, two providing the health talk and leading the group consent process, two administering the pre-test survey, and two administering the post-test survey. Personnel costs related to administering the pre-test and post-test surveys were not included in the costing model, because administering the surveys was an entirely research activity. A program administrator worked offsite to facilitate timely supply delivery. The CHCs each had 4 tent assemblers and 2 security guards hired on a per diem basis.

For notification, woman chose from four options: home visits, text messages, and phone calls. CHC staff provided participants with results within two weeks. Program assistants conducted text messages and phone calls. Home visits were led by the CHVs; CHVs conducting home visits would conduct a maximum of three attempts to provide results to the home. The program assistants documented the follow-up plan and outcomes in the notification logs. HPV-positive participants would receive standard referral to a treatment site, which was located at Migori County Hospital.

*Clinic Workflow*

In the clinic model, Outreach, Screening, and Notification phases occurred concurrently for the duration of the study (thirty-six weeks). The clinic CHVs employed by the program mobilized women to screen for cervical cancer through door-to-door mobilization and posters only (i.e. no public address systems and stakeholder meetings which were conducted at CHCs).

The clinic screening workflow was similar to the CHC except that a single CHV performed all steps: register the participants, facilitate group education and consent, administer the pre-test survey, direct participants to self-collection rooms, and administer the post-test survey. Two data clerks supervised the data collection process at clinics through weekly visits, at which time they also transported samples for processing at Migori County Hospital and ensured that CHVs were paid. The program coordinator oversaw the activities of the clinic-based screening and ensured supplies were adequate and that collection and testing of samples was timely. Similar to CHCs, the lab technician periodically visited clinics to perform quality control and ensure proper laboratory supplies management. The lab technician processed samples at Migori County Hospital. The program administrator facilitated the smooth operation of the clinics off-site by ensuring supplies and services were available on time. The data manager oversaw data clerks, ensured data quality, and trained CHVs on data tools.

Notification for clinics was similar to how notification was conducted at the CHCs. Women were able to choose from the same four options. Five program assistants were in charge of conducting text messages, phone calls, and home visits. The five program assistants conducted notification for each of the six clinics continuously throughout the nine months of screening; the total number of days spent on notification ranged from 10 to 15 days for each clinic across the nine months. Women screened who opted for clinic visits would walk into clinics and receive results. The program assistants would similarly document follow-up plans and outcomes. Each HPV-positive participant was also referred for treatment at Migori County Hospital.
