# Supplementary material for: Cost of HPV screening at community health campaigns (CHCs) and health clinics in rural Kenya
Source: BMC Health Serv Res. 2018 May 25;18:378. doi: 10.1186/s12913-018-3195-6 (PMC5970469; doi:10.1186/s12913-018-3195-6)
Supplement: Supplementary file 2 — Further detail on cost estimation methods of each cost type (personnel, capital, facility, recurrent goods, and services): Includes in-depth information on the sources, data collection method, and cost estimation method of each of the major cost category types. Includes how personnel costs were allocated across sites, and how capital costs were amortized. (DOCX 16 kb) [file 12913_2018_3195_MOESM2_ESM.docx]

**Additional file 2**

T.S. Section 2: Further detail on cost estimation methods of each cost type (personnel, capital, facility, recurrent goods, and services)

*Personnel Costs Estimation*

To estimate personnel costs, salaries information were extracted from salary records provided by the research coordinator. For CHCs, information on whether personnel time was dedicated to program or non-program activities during the screening phase were extracted from the Time-and-motion logs recorded by providers each day. Time-and-motion logs recorded at CHCs every day were converted into percentages of time each team member (i.e. provider) dedicated to screening activities (registration, informed consent, pre-test survey, collection of test kits, and post-test survey). The pre-test and post-test surveys were research activities, and thus were not costed. Providers recorded time spent on tasks throughout the day, and task categories included wait time, registration, group health education, and pre-survey administration, among others. For clinic personnel, the proportion of personnel time spent on program and non-program activities was based on interviews conducted with program staff.

Since clinic personnel worked on all six clinic communities concurrently, the total per-clinic personnel costs were estimated based on the number of women screened at each clinic, i.e. the proportion of women screened in each clinic community over the total number of women screened at clinics. For example, 157 women screened at God Jope, which is 7.7% of the total number of women screened at clinics (2042 women). God Jope was therefore assigned 7.7% percent of the total personnel costs at clinics. This method of personnel cost allocation was used for costing Screening and Notification activities. Outreach at clinics only involved CHVs (one CHV at each clinic) and thus did not require further allocation of salaries.

*Capital Goods and Facility Costs Estimation*

Capital goods are tangible assets including vehicle for transportation, tents for CHCs, and the *care*HPV ^TM^ test system, which is a rapid batch diagnostic test designed for HPV-testing in low-resources settings (Qiagen, Cost Gaithersburg, Maryland). Information on costs of capital goods was derived from expenditure records. For both CHC and clinic arms, interviews were also conducted with the program staff to verify the list of capital items, number of units used and allocation of items across program and non-program components. If the capital good was partially used for research purposes, a non-program component, a portion of the total cost of the good was not included in final per-screening cost calculations. Costs of capital items were amortized on a straight-line basis over five years assuming no salvage value. Each capital cost was also labeled with a “resource purpose”, which included overhead and administrative costs, training, community mobilization, transportation, counseling, and laboratory testing costs.

Facility costs from renting a space were only incurred by government health clinics. The facility cost was measured by multiplying the proportion of space at government health clinics dedicated to HPV-screening with MOH health facilities construction rates. For most clinics, HPV-screening occurred in a room shared with other services; the proportion of women participating in HPV screening over the total number of clients served in the room was calculated as an estimate of percentage of the space allocated to HPV-screening activities. For one clinic, the entire room was devoted to HPV screening services.

*Recurrent Goods and Services Costs Estimation*

Recurrent goods are goods that are not tangible assets. In the context of the program, recurrent goods include supplies such as the careHPV test kits, fuel costs, and lab supplies. Services are expenses from tasks performed by a person that was not a member of the study team. Cost estimates for recurrent goods and services were derived from expenditure records. For both CHC and clinic arms, interviews were conducted with the program staff to verify the list of recurrent items and services, number of units used, and allocation of items across different functions and phases of each program. Activity purposes included overhead and administrative costs, training, community mobilization, transportation, counseling, and laboratory testing costs.
