# Supplementary material for: Cost of HPV screening at community health campaigns (CHCs) and health clinics in rural Kenya
Source: BMC Health Serv Res. 2018 May 25;18:378. doi: 10.1186/s12913-018-3195-6 (PMC5970469; doi:10.1186/s12913-018-3195-6)
Supplement: Supplementary file 3 — Table S1. Major cost items for each cost type for CHCs, with data source information on quantity, unit and total costs, and % allocation across purposes. (DOCX 19 kb) [file 12913_2018_3195_MOESM3_ESM.docx]

**Additional file 3:** Table S1. Major cost items for each cost type for CHCs, with data source information on quantity, unit and total costs, and % allocation across purposes

| Cost Type | Specific Cost | Source of Quantity Estimation (# units) | Source of Price | Source of % Allocation across purposes |
| --- | --- | --- | --- | --- |
| [1] | [2] | [3] | [4] | [6] |
| Capital Goods | Transport Vehicle | WT | ER | WT |
| (+Facility) | 2 tents | I | ER | H |
|  | careHPV Test system | I | ER | H |
|  | Chairs-plastic | I | ER | H |
|  | Power Bank | I | I | H |
|  | 6 Tables | I | ER | H |
|  | Pipetman F50 50µl | I | ER | H |
|  | Repeater pipette | I | ER | H |
|  | UPS 1000VA APC Smart | I | ER | H |
|  | Cooler boxes-11 litres | I | I | H |
|  | Sample racks | I | MAR | H |
|  |  |  |  |  |
| Recurrent Goods | Fuel cost | WT | I | WT |
|  | careHPV Test Kits CE (96-test) | I | ER | H |
|  | careHPV Collection Medium | I | ER | H |
|  | careHPV Brush | I | ER | H |
|  | Pipette tips | I | ER | H |
|  | Cleaning clothes | I | ER | H |
|  | T-shirts | I | ER | H |
|  | Staff refreshments | I | I | H |
|  | Brochures | I | ER | H |
|  | Gloves | I | ER | H |
|  | Plate sealers | I | ER | H |
|  | Foam specimen tube rack | I | ER | H |
|  | Tripple Timers | I | ER | H |
|  | Barcodes | I | I | H |
|  | Sanitizer | I | ER | H |
|  | Biohazard bags | I | I | H |
|  | Specimen tracking log | I | I | H |
|  | Tissue | I | I | H |
|  | Stamp pad | I | I | H |
|  | Box files- plastic globe | C | I | H |
|  | Consent forms | I | I | H |
|  | Education module | I | ER | H |
|  | Pens | I | ER | H |
|  | Recruitment scripts | I | ER | H |
|  |  |  |  |  |
| Services | Public address system | I | I | H |
|  | CHW Mobilization | I | I | H |
|  | Pick-up Hire | I | I | H |
|  | Puncture repair | I | I | H |
|  | Tent repair, cleaning and loading | I | I | H |
|  | Flip charts for training | I | I | H |
|  | Transport reimbursement | I | I | H |
|  | Transport reimbursement for Home visits | I | I | H |
|  | Airtime for Phone calls | I | I | H |
|  | Mobile handset for phone calls | I | ER | H |
|  |  |  |  |  |
| Personnel | Program Coordinator | C | PAY | I |
|  | Costing Lead | C | PAY | A |
|  | Program Administrator | C | PAY | I |
|  | Data Manager | C | PAY | I |
|  | Assistant Data Manager | C | PAY | I |
|  | Program Assistant | C | PAY | TM |
|  | Lab Technician | C | PAY | I |
|  | Study driver | C | PAY | A |
|  | 10 CHVs | I | I | A |
|  | 4 tent assemblers | I | I | A |
|  | 2 security | I | I | A |

Legend:

WT = Work Ticket (document used for transport management; documents particulars of a journey)

A = Approximation made by the costing lead

C = Direct count, by costing lead

H = 100 percent allocation to program

I = interviews conducted with program staff

PAY = salary records provided by research coordinator (KEMRI CCSP Study Payroll)

ER = expenditure records

MOH= NASCOP/MOH data of approximate cost of facility set up

MAR=Market rates

TM = provider time and motion data
