# Supplementary material for: Cost of HPV screening at community health campaigns (CHCs) and health clinics in rural Kenya
Source: BMC Health Serv Res. 2018 May 25;18:378. doi: 10.1186/s12913-018-3195-6 (PMC5970469; doi:10.1186/s12913-018-3195-6)
Supplement: Supplementary file 4 — Table S2. Major cost items for each cost type for Clinics, with data source information on quantity, unit and total costs, and % allocation across purposes. (DOCX 18 kb) [file 12913_2018_3195_MOESM4_ESM.docx]

**Additional file 4:** Table S2. Major cost items for each cost type for Clinics, with data source information on quantity, unit and total costs, and % allocation across purposes

| Cost Type | Specific Cost | Source of Quantity Estimation (# units) | Source of Price | Source of % Allocation across purposes |
| --- | --- | --- | --- | --- |
| Capital Goods | Facility space | C | MOH | I |
| (+Facility) | careHPV test system | I | ER | H |
|  | Motor bike | C | I | I |
|  | careHPV Test system | I | ER | H |
|  | Pipetman F50 50µl | I | ER | H |
|  | Repeater pipette | I | ER | H |
|  | UPS 1000VA APC Smart | I | ER | H |
|  | Cooler boxes-11 litres | I | I | H |
|  | Sample racks | I | MAR | H |
|  |  |  |  |  |
| Recurrent Goods | Recruitment scripts | I | I | H |
|  | careHPV Test Kits CE (96-test) | I | ER | H |
|  | careHPV Collection Medium | I | ER | H |
|  | careHPV Brush | I | ER | H |
|  | Pipette tips | I | ER | H |
|  | Tripple Timers | I | ER | H |
|  | Brochures | I | ER | H |
|  | Barcodes | I | I | H |
|  | Sanitizer | C | ER | H |
|  | Biohazard bags | C | I | H |
|  | Specimen tracking log | C | I | H |
|  | Tissue | C | I | H |
|  | Stamp pad | C | I | H |
|  | Box files- plastic globe | C | I | H |
|  | Consent forms | I | I | H |
|  | Education module | C | ER | H |
|  | Pens | I | I | H |
|  | Clear bags | I | ER | H |
|  | Cleaning clothes | I | ER | H |
|  | Gloves | I | ER | H |
|  | Plate sealers | I | ER | H |
|  | Foam specimen tube rack | I | ER | H |
|  |  |  |  |  |
| Services | Flip charts for training | I | ER | H |
|  | Transport reimbursement | I | I | H |
|  | Transport reimbursement for Home visits | I | I | H |
|  | Airtime for Phone calls | I | I | H |
|  | Mobile handset for phone calls | I | ER | H |
|  |  |  |  |  |
| Personnel | Program Coordinator | C | PAY | I |
|  | Costing lead | C | PAY | A |
|  | Costing assistant | C | PAY | I |
|  | Data Manager | C | PAY | I |
|  | Assistant data manager | C | PAY | I |
|  | Program Assistants | C | PAY | I |
|  | Lab Technician | C | PAY | I |
|  | 2 Data clerks | C | PAY | I |
|  | 10 CHV (100% of 9 months) | I | I | A |
|  | Program administrator | C | PAY | I |
|  |  |  |  |  |

Legend:

WT = Work Ticket (document used for transport management; documents particulars of a journey)

A = Approximation made by the costing lead

C = Direct count, by costing lead

H = 100 percent allocation to program

I = interviews conducted with program staff

PAY = salary records provided by research coordinator (KEMRI CCSP Study Payroll)

ER = expenditure records

MOH= NASCOP/MOH data of approximate cost of facility set up

MAR=Market rates

TM = provider time and motion data
