# Supplementary material for: Cost of HPV screening at community health campaigns (CHCs) and health clinics in rural Kenya
Source: BMC Health Serv Res. 2018 May 25;18:378. doi: 10.1186/s12913-018-3195-6 (PMC5970469; doi:10.1186/s12913-018-3195-6)
Supplement: Supplementary file 5 — Table S3. Cost estimations per woman screened at all 10 clinics, from January to September 2016. Includes cost estimations of the three phases of implementation: outreach and mobilization, screening, and notification and standard referral. (DOCX 22 kb) [file 12913_2018_3195_MOESM5_ESM.docx]

**Additional file 5: Table S3**. Cost estimations per woman screened at all 10 clinics, from January to September 2016

|  | **Clinic #** | | | | | | | | | |
| --- | --- | --- | --- | --- | --- | --- | --- | --- | --- | --- |
|  | **1** | **2** | **3** | **4** | **5** | **6** | **7** | **8** | **9** | **10** |
| Outreach and Mobilization |  |  |  |  |  |  |  |  |  |  |
| Personnel | 0.15 | 0.20 | 0.23 | 0.10 | 0.12 | 0.15 | 0.13 | 0.20 | 0.18 | 0.20 |
| Recurrent Goods | 0.02 | 0.02 | 0.02 | 0.01 | 0.01 | 0.02 | 0.01 | 0.02 | 0.02 | 0.02 |
|  |  |  |  |  |  |  |  |  |  |  |
| Screening |  |  |  |  |  |  |  |  |  |  |
| Capital Goods | 1.03 | 1.03 | 1.04 | 0.99 | 1.00 | 1.50 | 1.00 | 1.03 | 1.02 | 1.67 |
| Facility | 2.49 | 2.09 | 6.03 | 10.78 | 0.41 | 0.48 | 1.90 | 2.06 | 4.94 | 1.52 |
| Personnel | 12.92 | 13.20 | 12.92 | 13.63 | 13.20 | 13.20 | 13.20 | 13.20 | 13.20 | 12.97 |
| Recurrent Goods | 5.70 | 7.58 | 7.14 | 5.96 | 6.77 | 6.10 | 6.51 | 6.46 | 6.15 | 6.74 |
| Services | 1.98 | 2.82 | 2.44 | 1.70 | 1.55 | 1.36 | 1.72 | 2.59 | 3.02 | 1.46 |
|  |  |  |  |  |  |  |  |  |  |  |
| Notification and Standard Referral |  |  |  |  |  |  |  |  |  |  |
| Personnel | 10.46 | 10.46 | 10.46 | 10.46 | 10.46 | 10.46 | 10.46 | 10.46 | 10.46 | 10.46 |
| Services | 0.09 |  | 0.47 | 0.71 |  |  |  |  |  |  |
|  |  |  |  |  |  |  |  |  |  |  |
| # women screened | 211 | 157 | 137 | 326 | 269 | 209 | 239 | 156 | 178 | 160 |
| Cost per screening | 34.83 | 37.39 | 40.76 | 44.34 | 33.52 | 33.27 | 34.93 | 36.02 | 38.98 | 35.05 |
